# Supplementary figures and images for: Exome-wide association study reveals largely distinct gene sets underlying specific resistance to dengue virus types 1 and 3 in Aedes aegypti
Source: PLoS Genet. 2020 May 28;16(5):e1008794. doi: 10.1371/journal.pgen.1008794 (PMC7282673; doi:10.1371/journal.pgen.1008794)

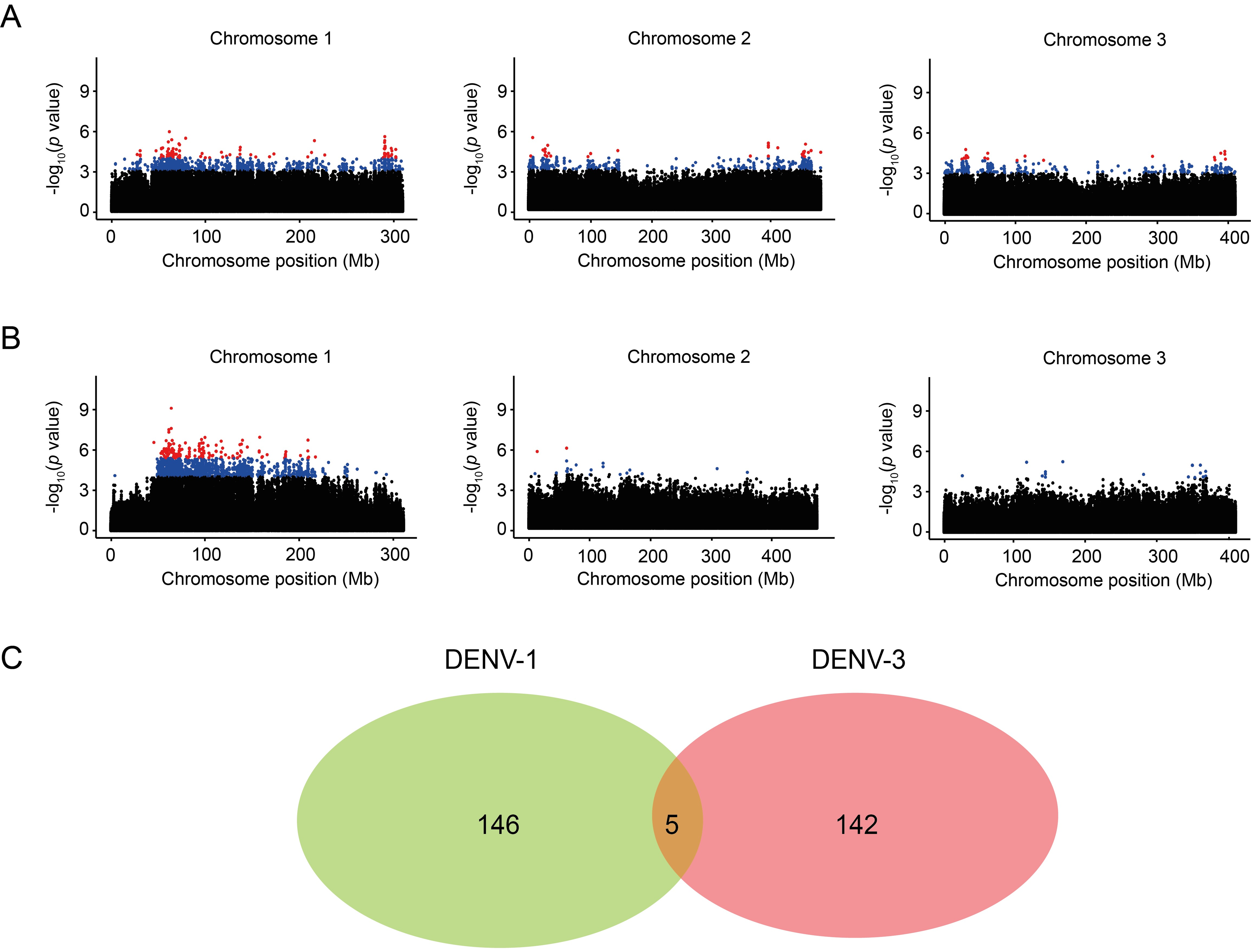

Supplement: S1 Fig — (A) Manhattan plot of p values representing the association between SNP frequency and DENV-1 resistance, distributed along the three chromosomes. SNP frequency was compared between phenotypic pools of mosquitoes that are either resistant of susceptible to DENV-1. (B) Manhattan plot of p values representing the association between SNP frequency and DENV-3 resistance, distributed along the three chromosomes. SNP frequency was compared between phenotypic pools of mosquitoes that are either resistant of susceptible to DENV-3. In (A) and (B), each dot represents a single SNP and is colored according to the statistical significance of the genotype-phenotype association. Blue dots represent the lower 5% of p values and red dots represent the lower 2.5% p values. (C) Venn diagram of the 0.001% most significant SNPs associated with DENV-1 infection, DENV-3 infection, or both. (TIF) [file pgen.1008794.s001.tif]

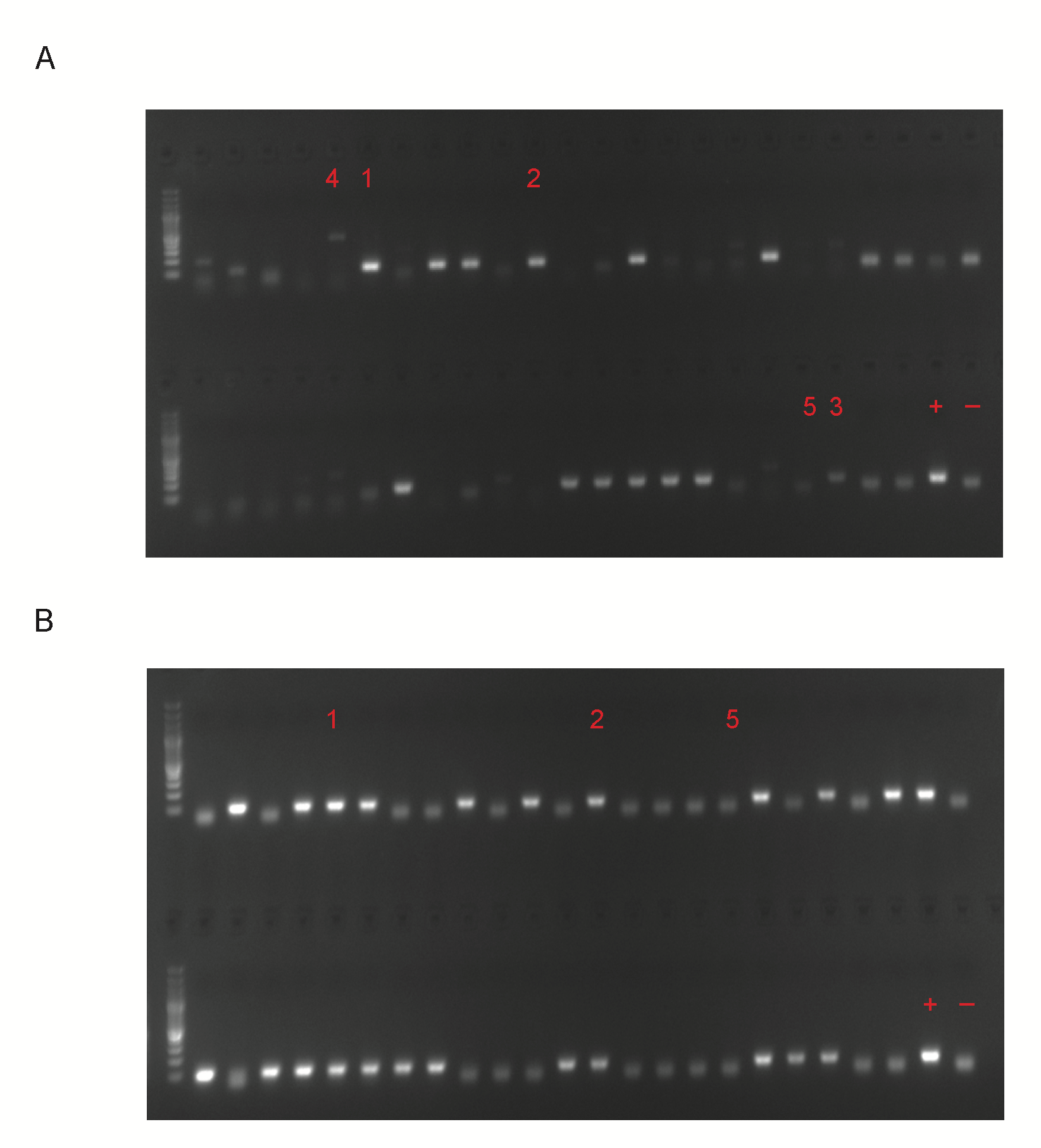

Supplement: S2 Fig — Photos of representative electrophoresis gels of RT-PCR products for DENV-1 (A) and DENV-3 (B) detection in mosquito bodies. The readout was based on five scores (exemplified in red font) as follows: 1 = clear and bright band at the right height; 2 = clear and moderately bright band at the right height; 3 = weak band at the right height; 4 = one or several bands at an unexpected height (sometimes accompanied by the right band); 5 = no band. A sample was only considered DENV-positive when its score was 1 or 2. For DENV-1 all five scores were typically present on the gels, whereas for DENV-3 scores 3 and 4 were typically absent. The + and–symbols denote positive and negative controls, respectively. (TIF) [file pgen.1008794.s002.tif]
