# Supplementary material for: Exome-wide association study reveals largely distinct gene sets underlying specific resistance to dengue virus types 1 and 3 in Aedes aegypti
Source: PLoS Genet. 2020 May 28;16(5):e1008794. doi: 10.1371/journal.pgen.1008794 (PMC7282673; doi:10.1371/journal.pgen.1008794)
Supplement: Attachment — Submitted filename: Dickson_response_to_reviewers.pdf [file pgen.1008794.s006.pdf]

Institut Pasteur

Paris, 7th of April 2020

Dear Prof. Sirugo,

Thank you for your correspondence about our manuscript PGENETICS-D-20-00228 entitled 'Exome-wide association study reveals largely distinct gene sets underlying specific resistance to dengue virus type 1 and 3 in *Aedes aegypti*'. We are submitting a new version of our manuscript in which we addressed the Reviewers' comments. The changes are detailed in the point-by-point response below, in which the Reviewers' comments are in blue font and our responses are in black font. Lines numbers refer to the version of the manuscript with track changes.

We are grateful to the reviewers for their insights, which contributed to greatly improve the manuscript.

Please let us know if you need any additional information.

Thank you very much for your consideration.

Sincerely,

Louis Lambrechts

Head, Insect-Virus Interactions Unit
Department of Virology
Institut Pasteur – CNRS UMR 2000
28 rue du Docteur Roux
75724 Paris Cedex 15 France

Reviewer #1: The study by Dickson and colleagues report that different sets of genes condition the refractory phenotype of Aedes aegypti to dengue virus (DENV) serotype 1 and 3. The authors conducted exome sequencing of mosquito pools showing differential interaction with the two serotypes, and applied a SNP based association statistics to predict the host genes that likely controlled the observed phenotypes. I find the study interesting but highly intriguing. Here are the problems of this study-

Major Issues

1. The central hypothesis of this study is that if a vector population responds differentially to two serotypes, then factors associated with the host could explain that phenotypic variation (lines 165-167). But, the observed resistance phenotype could also be due to factors associated with the virus. What was done to rule out that possibility?

Response: We thank the Reviewer for this important comment and we apologize if it was unclear. By definition, a G x G interaction means that both virus and host factors are at play. In other words, if mosquito phenotypic variation was entirely explained by virus factors, we would expect the Cairns mosquito population to display the same serotype-specific response as the Bakoumba population. In this study we focused on host factors but we certainly did not rule out the existence of virus factors. Virus factors were simply out of the scope of this study.

To address this point we added the following sentence on lines 316-318: "Note that in this study, we focused on the host factors involved in the G x G interaction but the virus factors remain to be elucidated."

2. The major premise of this study also lack the very fact that genetic changes in the non-coding regions of genome are major contributors of gene regulation. It would make better sense if the authors had sequenced the whole genome, not the exome alone, to identify the associated SNPs. To me, this is a major flaw in the experimental design relative to the said objective of the study.

Response: We agree with the Reviewer that variation in non-coding regions could contribute to phenotypic variation. Our approach relied on the assumption that coding sequences were a faithful representation of the genome. In the absence of significant genotype-phenotype association the results would have been inconclusive, however because we did identify different exome variants associated with DENV-1 and DENV-3 resistance this was sufficient to support our conclusion that the genetic basis of DENV-1 resistance is distinct from the genetic basis of DENV-3 resistance in this mosquito population.

To address this point we added the following sentence on lines 331-334: "Nevertheless, detection of different exome variants associated with resistance to DENV-1 and resistance to DENV-3 is sufficient to support our conclusion that their respective genetic architecture is distinct."

3. The authors identified two sets of non-overlapping genes that condition resistance exclusively to DENV1 vs DENV3. To accept that result, it is necessary to provide data that those genes differentially respond to infection with DENV1 vs DENV3. To show that the authors need to provide the expression level of genes, may be in the dissected midguts of the females, after infecting with the two serotypes.

Response: Although we acknowledge that it would be interesting to determine whether candidate genes also display allelic differences in gene expression, with all due respect we disagree with the Reviewer that this investigation is necessary to support our conclusions. A genetic association does not imply differential expression, that is, different alleles of the same gene can confer a different phenotype even if their level of expression is similar. Perhaps one of the best examples to illustrate this point is the abnormal S allele of the hemoglobin beta gene, which confers protection against severe malaria in the absence of differential expression relative to the normal A allele (the two alleles are codominant with respect to the actual blood concentration of hemoglobin).

To address this point we added the following sentence on lines 365-367: "Although a genetic association does not necessarily imply differential expression, it would be interesting to determine whether candidate genes also display allelic differences in gene expression."

Minor issue

The discussion is largely descriptive. The authors should explain what is the biological meaning of their findings. What are the functional and evolutionary implication that a vector mosquito must utilize non-overlapping gene sets to defend infection by DENV serotypes. Is there an evolutionary benefit for the vector? Is it relevant (the host effect) to differential prevalence of DENV1 relative to DENV3?

Response: Following the Reviewer's suggestion, we added a paragraph to the discussion to speculate on the evolutionary implications of non-overlapping sets of genes underlying DENV type-specific resistance in mosquitoes (lines 383-388):

"Although the forces driving the evolution of DENV resistance in *Ae. aegypti* are largely unknown, one evolutionary implication of the distinct gene set underlying resistance to DENV-1 and DENV-3 is that the evolution of resistance to one DENV type is not expected to lead to the correlated evolution of resistance to another DENV type. This finding is consistent with the absence of virus cross-resistance (Cogni et al. 2016) and the lack of genetic trade-offs between the levels of resistance to different viral genotypes (Carpenter et al. 2012) in *Drosophila*."

To address the epidemiological relevance of our findings, we added the following paragraph to the discussion (lines 390-398):

"The epidemiological relevance of our results is difficult to assess because dengue epidemiology is poorly documented in Gabon. A recent study reported DENV-3 circulation in 2016-2017 (Abe et al. 2020) whereas previous dengue outbreaks were mainly associated with DENV-2 in 2007 and in 2010 (Caron et al. 2013), but this information is insufficient to

make a link between the DENV-1 resistance phenotype of the Bakoumba population and the relative lack of this DENV type in recently reported outbreaks in Gabon. In our experiments, we used a DENV-1 isolate from Thailand and a DENV-3 isolate from Gabon but the geographical origin of the virus is unlikely to have influenced the results due to the lack of evidence for DENV local adaptation to *Ae. aegypti* populations (Fansiri et al. 2016)."

Reviewer #2: Here, the authors have produced a paper investigating the genetic architecture of dengue virus (DENV) resistance in a population of Aedes aegpti from Bakoumba, Gabon. This population displays a stronger resistance phenotype to DENV-1 compared to DENV-3. The authors used experimental bloodmeal exposures and exome sequencing of large phenotypic pools that were either susceptible or resistant. The paper is well written but I believe there are several things that need to be addressed to facilitate understanding by the reader.

First, the authors demonstrate that their Bakoumba population of Ae. aegypti have differential susceptibility to DENV-1 vs. DENV-3 and that this is likely the result of mosquito genetic factors because a population of Ae. aegypti from Cairns, Australia were equally susceptible to DENV-1 and DENV-3. While I do not expect the authors to do additional experiments, I do wonder why there was no comparison between the two mosquito populations? This seems like an ideal way to validate susceptibility loci in the Bakoumba population.

Response: We did not perform a direct comparison between the Bakoumba and the Cairns populations because the high level of genetic differentiation between them would have likely prevented a meaningful conclusion. The Bakoumba population presumably belongs to the African subspecies, *Aedes aegypti formosus*, whereas the Cairns population presumably belongs to the globally invasive subspecies, *Aedes aegypti aegypti*, therefore the two populations are expected to be highly genetically divergent (Gloria-Soria et al. Mol Ecol 2016). In other words, any loci underlying DENV resistance that are shared or divergent between the two populations would have been masked by the overwhelming amount of genetic differences overall. In contrast, the comparison between phenotypically resistant or susceptible individuals within the same population (Bakoumba) allowed us to control for the genetic background.

To address this point we added the following paragraph to the results section on lines 163-170: "A direct comparison between the Bakoumba and the Cairns populations would be impractical because their genetic differentiation would likely mask any loci underlying DENV resistance that are shared or divergent between them. Indeed, the Bakoumba population presumably belongs to the African subspecies, *Ae. aegypti formosus*, whereas the Cairns population presumably belongs to the globally invasive subspecies, *Ae. aegypti aegypti*, therefore the two populations are expected to be genetically divergent (Gloria-Soria et al. 2016). In contrast, the comparison between phenotypically resistant or susceptible individuals within the Bakoumba population allowed us to control for the genetic background."

Second, it would be informative to know DENV epidemiological data for Bakoumba, Gabon. Critically, have there been outbreaks of DENV-1? The authors used a DENV-1 isolate from Thailand for experiments, whereas the DENV-3 isolate was from Gabon. This is somewhat controlled with the Cairns vector competence experiments, but it does raise the question of the appropriateness of the DENV-1 isolate for GxG experiments.

Response: Dengue epidemiology is poorly documented in Gabon but a recent study reported DENV-3 circulation in 2016-2017 (Abe et al. Int J Infect Dis 2020) whereas previous dengue outbreaks were mainly associated with DENV-2 in 2007 and in 2010 (Caron et al. PLoS One 2013). The available information is insufficient to make a link between the susceptibility profile of only one Gabonese population and this epidemiological pattern. To our knowledge there is no specific information on dengue in Bakoumba village.

To address this point, we added the following paragraph to the discussion (lines 390-395):

"The epidemiological relevance of our results is difficult to assess because dengue epidemiology is poorly documented in Gabon. A recent study reported DENV-3 circulation in 2016-2017 (Abe et al. 2020) whereas previous dengue outbreaks were mainly associated with DENV-2 in 2007 and in 2010 (Caron et al. 2013), but this information is insufficient to make a link between the DENV-1 resistance phenotype of the Bakoumba population and the relative lack of this DENV type in recently reported outbreaks in Gabon."

Because DENV-1 has not been widely circulating in Gabon, we did not have a DENV-1 isolate from Gabon at our disposal. However, we observed that the Bakoumba population was equally susceptible to the DENV-3 isolate from Gabon and to a DENV-3 isolate from Thailand (see prevalence data shown in the figure below, provided only for the Reviewer's eyes). This observation and a previous study (Fansiri et al. Evol Appl 2016) are consistent with a lack of local adaptation between *Ae. aegypti* and DENV.

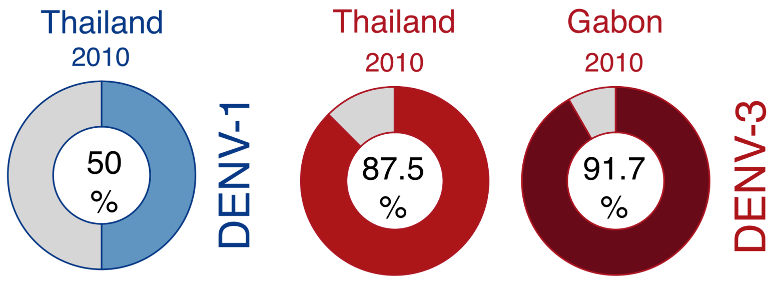

To address this point, we added the following sentence to the discussion (lines 395-398):

"In our experiments, we used a DENV-1 isolate from Thailand and a DENV-3 isolate from Gabon but the geographical origin of the virus is unlikely to have influenced the results due to the lack of evidence for DENV local adaptation to *Ae. aegypti* populations (Fansiri et al. 2016)."

Third, as I understand it, exome sequencing was done from mosquito heads only. Given the tissue-specific nature of resistance mechanisms in different host-pathogen combinations, is there potential for information to be missed when not using the whole body?

Response: Exome sequencing was performed on the heads because the DNA extraction procedure is different from the virus detection protocol (RNA extraction and RT-PCR). DNA was extracted from the head for exome sequencing so that the body remainder would be available for phenotypic characterization (virus detection). There is no reason to believe that genomic DNA from the head is different from genomic DNA in the rest of the body.

Reviewer #3: This is a very interesting and well-written paper investigating how differential SNP frequencies in the exome of an Aedes aegypti population from Gabon (affecting different gene sets) account for variable resistance levels to dengue 1 and dengue 3 viruses. The mosquito population is significantly more resistant to DENV1 than to DENV3. Mosquitoes were exposed to various doses of either virus followed by selection for extremely DENV3 susceptible/resistant versus DENV1 susceptible/resistant phenotypes. Once selected, they were subjected to whole-exome sequencing. Based on computational differential SNP analysis, the authors revealed that resistance to DENV1 in the Gabon population was largely based on different gene sets than resistance to DENV3.
This work is important as it shows on a global level how gene polymorphisms in Ae. aegypti, an organism with a highly complex genome structure, contribute to pathogen resistance phenotypes. The experimental design has been carefully chosen and the data analysis looks thorough.

There are a few important issues that need to be addressed.

Abstract:
Line 38: ….the exomes of…..

Response: We made this correction.

Introduction:
Line 96: …population in the long term….

Response: We apologize if we misunderstand the Reviewer's suggestion but we purposely used the plural form for mosquito populations.

Line 114: how do you define here strongly resistant versus moderately resistant?

Response: We quantified the level of resistance based on the dose-response experiments present in the results section (Fig. 1A). We arbitrarily use "strongly resistant" and "moderately resistant" to denote a 50% oral infectious dose above and below 6 $\log_{10}$ FFUs/mL, respectively. To address this point we modified the sentence as follows:

"Using dose-response experiments, we found the Bakoumba population to be strongly resistant to DENV-1 and only moderately resistant to DENV-3 infection."

Line 126: …gene-wide….

Response: We made this correction.

Results:

The Results section should be subdivided using sub-headers for the different paragraphs.

Response: We added four sub-headings in the results section.

Line 189 onwards: This is not very clear to the reader - what does 'individually phenotyped' mean, testing of carcasses via RT-PCR for the presence/absence of virus?? "DNA was extracted from 182, 174, 176 females…..(carcasses/head tissues??); …..combined into 12 standardized phenotypic pools of 30-48 individuals." What does standardized mean here? What individuals? This all should be made more clear using precise descriptions because this information is crucial to understand the experimental design und ultimately the results.

Response: We clarified this paragraph as follows:

"The body of 668, 690 and 680 females was individually tested for DENV infection by RT-PCR in replicate experiments 1, 2 and 3, respectively (Table 3). Based on their resistant or susceptible phenotype, 182, 174 and 176 females were selected for DNA extraction from experiments 1, 2 and 3, respectively. DNA extracted from the head of individual females was combined into 12 phenotypic pools (3 experiments x 2 DENV types x 2 phenotypes) of 30-48 individuals (mean 44.3) to prepare 12 libraries for exome sequencing (Table 3). Individual DNA concentrations were adjusted prior to pooling so that each individual contributed the same amount of DNA to the library."

Table 3: "…tested DENV-positive or DENV-negative…"perhaps add here "based on RT-PCR results"

Response: We modified the sentence as follows:

"The table shows the number of Bakoumba mosquitoes that tested DENV-positive or DENV-negative by RT-PCR (Figure S2) in each of the experimental conditions of the screen."

Line 218: is there a reason for using 2 different genome assembly/annotation versions? The analysis still could have been done using version 3 instead of 5? Would this likely/possibly affect the results? A comment here would be helpful.

Response: The probes for exome capture were designed when only AaegL3 was available. The analysis was performed with AaegL5 because the gene annotation is significantly better (Matthews et al. Nature 2018). The difference is not expected to affect our results as long as the same analysis is applied to the phenotypic pools (resistant and susceptible). We initially ran the analysis with AaegL3 and made the same conclusions. To address this point we modified the sentence as follows:

"Note that although the exome capture probes were designed using the AaegL3 genome build (Nene et al. 2007) because it was the only one available at the time, our gene-based analysis was performed using the more recent AaegL5 genome build (Matthews et al. 2018), which is significantly better annotated."

Additional comment: since virus resistance levels of the “exosome samples” were assessed by RT-PCR, perhaps it would be a good idea to include a representative gel image into the supplemental information showing various PCR amplicons for DENV1 and DENV3 and also positive/negative controls. When a sample was considered negative, was there absolutely no signal at all? What about a very faint signal? How was this classified?

Response: According to the Reviewer’s suggestion, we added a new Figure S2 providing representative pictures of the electrophoresis gels and a rationale for scoring RT-PCR results. The readout was based on five scores as follows: 1 = clear and bright band at the right height; 2 = clear and moderately bright band at the right height; 3 = weak band at the right height; 4 = one or several bands at an unexpected height (sometimes accompanied by the right band); 5 = no band. A sample was only considered DENV-positive when its score was 1 or 2. For DENV-1 all five scores were typically present on the gels, whereas for DENV-3 scores 3 and 4 were typically absent.

Discussion:
Perhaps also add some discussion points about possible functions of genes with zinc binding activity and those with ATP binding activity / sulfur compound transmembrane transporter activity in the context of mosquito infection with DENV.

Response: Following the Reviewer’s suggestion, we added a paragraph in the discussion to speculate on the possible function of genes with zinc binding activity, ATP binding activity and sulfur compound transmembrane transporter activity in the context of mosquito infection by DENV (lines 369-380):

“Mosquito genes uniquely associated with resistance to DENV-3 infection were enriched in genes with zinc ion binding activity, whereas genes associated with resistance to both DENV-1 and DENV-3 infection were enriched in genes with ATP binding activity and sulfur compound transmembrane transporter activity. Zinc is an essential cofactor that ensures the proper folding and functioning of not only cellular proteins but also viral proteins (Lazarczyk et al. 2008). To our knowledge, there is no prior evidence for a link between zinc ion binding activity and mosquito-virus interactions, however host cellular systems controlling zinc balance are known to interfere with virus replication (Lazarczyk et al. 2008). Likewise, ATP binding activity and sulfur compound transmembrane transporter activity have not been specifically reported to participate in mosquito-virus interactions, however the high dependence of viruses on the cellular machinery makes any molecular function potentially relevant to host-virus interactions.”

Line 288:….about 2% of the entire……

Response: We made this correction.

Line 301: …in genetic ascertainment because for…. (delete ‘in’).

Response: We made this correction.

Line 305: change “Thus” for “This”

Response: We made this correction.

Line 312: ….surrounded by a large region…..

Response: We made this correction.

Materials and Methods:
Describe, how low and high DENV doses were prepared, validated, and administered to mosquitoes.

Response: To address this point we added the following sentence (lines 447-449):

"To expose mosquitoes to different virus concentrations in the dose-response experiments, virus titer in the blood meal was adjusted prior to its preparation by diluting the virus stock in cell culture medium."

Line 345 & 347: replace "derived" for "obtained"

Response: We made this correction.

Line 375: …..was synthetized using random hexamers…..5 ul of lysate was included in a 20 ul reaction, following…..

Response: We made these corrections.

Line 412: this is not clear: "…..equal amounts of DNA from each individual were pooled to reach the required 1.0 ng of DNA required for library preparation." Please re-phrase/clarify.

Response: We clarified this section as follows:

"To prepare the 12 sequencing libraries (2 DENV types x 2 phenotypes x 3 replicate experiments), individual DNA samples were combined to obtain 1.0 ng of DNA per pool. Individual DNA concentrations were adjusted prior to pooling so that each individual contributed the same amount of DNA to the pool."

Figure legends:
Line 702:….at 10 days post….

Response: We made this correction.

Figures:
Figure 1 and experiment referring to figure 1: two separate experiments were conducted in which Gabon and Australia mosquitoes were exposed to 3 different infectious doses ranging from 104 to 107 FFU/ml using DENV1 and DENV3. This experimental setup is difficult to retrieve from Fig. 1A. Where/how are the 3 different infectious doses shown? Based on that description, 24 data points should be shown instead of those 22 data points being presented. This is confusing. Perhaps the graph should be separated into two graphs: one for experiment 1 and another one for experiment 2.

Response: The infectious doses are shown on the x-axis. For each experiment there are three doses for each virus. The three doses of experiment 1 are covering a lower range (4 to 6.5 log_{10} FFUs/mL) than the three doses of experiment 2 (5.5 to 7.5 log_{10} FFUs/mL), which is why

feel that the two experiments should remain combined to comprehensively represent the dose response. Only 23 out of 24 data points are apparent because one of them (Bakoumba DENV-3 experiment 2) is masked due to the overlap with another value at 100% (Cairns DENV-3 experiment 2). Two other data points (Bakoumba DENV-1 experiment 1 and Cairns DENV-1 experiment 1) are superimposed at 0% but because the symbols are open the reader can see them both.

To address this point we modified the Figure 1 legend as follows:

"(**A**) Dose-response curves of *Ae. aegypti* colonies from Bakoumba (Gabon) or Cairns (Australia) challenged with DENV-1 and DENV-3. The percentage of DENV-infected mosquitoes at 10 days post exposure is shown as a function of the blood meal titer in $\log_{10}$-transformed focus-forming units (FFUs)/mL. The data was obtained in two separate dose-response experiments with three doses for each virus. The three doses of experiment 1 are covering a lower range than the three doses of experiment 2. Curves are logistic regressions of the data with their 95% confidence intervals indicated by shaded bands. Note that one data point (Bakoumba DENV-3 experiment 2) is masked due to the overlap with another value at 100% (Cairns DENV-3 experiment 2)."

Furthermore, the legend, open closed circles for experiment 1 & 2 and large, small circles for different N are way too tiny.

Response: We increased the circle size in the legend.

Figure 2: perhaps it would be helpful to extent the figure showing a complete experimental flow chart including number of mosquito carcasses tested for virus titers (FFU/ml), number of replicates, number of head tissues collected, pooling strategy of head tissue samples, and information on sequencing/data analysis strategy.

Response: All of the requested information is already provided in Table 3 so we strongly feel that an additional flowchart in Figure 2 would make it unnecessarily busy.
